# Preseason shoulder range of motion screening and in-season risk of shoulder and elbow injuries in overhead athletes: systematic review and meta-analysis

**DOI:** 10.1136/bjsports-2019-100698

**Published:** 2020-01-14

**Authors:** Federico Pozzi, Hillary A Plummer, Ellen Shanley, Charles A Thigpen, Chase Bauer, Melissa L Wilson, Lori A Michener

**Affiliations:** 1 Department of Physical Therapy, University of Florida, Gainesville, Florida, USA; 2 Division of Biokinesiology and Physical Therapy, University of Southern California, Los Angeles, California, USA; 3 Andrews Research & Education Foundation, Gulf Breeze, Florida, USA; 4 ATI Physical Therapy, Greenville, South Carolina, USA; 5 Western University of Health Sciences, Pomona, California, USA; 6 Department of Preventive Medicine, University of Southern California, Los Angeles, California, USA

**Keywords:** baseball, handball, swimming

## Abstract

**Objective:**

To characterise whether preseason screening of shoulder range of motion (ROM) is associated with the risk of shoulder and elbow injuries in overhead athletes.

**Design:**

Systematic review and meta-analysis.

**Data sources:**

Six electronic databases up to 22 September 2018.

**Eligibility criteria:**

Inclusion criteria were (1) overhead athletes from Olympic or college sports, (2) preseason measures of shoulder ROM, (3) tracked in-season injuries at the shoulder and elbow, and (4) prospective cohort design. Exclusion criteria were (1) included contact injuries, (2) lower extremity, spine and hand injuries, and (3) full report not published in English.

**Results:**

Fifteen studies were identified, and they included 3314 overhead athletes (baseball (74.6%), softball (3.1%), handball (16.1%), tennis (2.0%), volleyball (2.0%) and swimming (2.2%)). Female athletes are unrepresented (12% of the overall sample). Study quality ranged from 11 to 18 points on a modified Downs and Black checklist (maximum score 21, better quality). In one study, swimmers with low (<93°) or high (>100°) shoulder external rotation were at higher risk of injuries. Using data pooled from three studies of professional baseball pitchers, we showed in the meta-analysis that shoulder external rotation insufficiency (throwing arm <5° greater than the non-throwing arm) was associated with injury (odds ratio=1.90, 95% confidence interval 1.24 to 2.92, p<0.01).

**Conclusion:**

Preseason screening of shoulder external rotation ROM may identify professional baseball pitchers and swimmers at risk of injury. Shoulder ROM screening may not be effective to identify handball, softball, volleyball and tennis players at risk of injuries. The results of this systematic review and meta-analysis should be interpreted with caution due to the limited number of studies and their high degree of heterogeneity.

**PROSPERO registration number:**

CRD42017072895.

## Introduction

Overuse shoulder and elbow injuries are common across different overhead athletes regardless of age, sex and level of playing.^1–5^ Evaluating potential environmental-specific (extrinsic) and individual-specific (intrinsic) risk factors for shoulder and elbow injuries in overhead athletes is a research priority. Extrinsic risk factors include sport specialisation, training intensity, number of games per week, and number of pitches or throws per game and over a year.^6–10^ Extrinsic factors may contribute to overuse injuries due to repetitive load on the shoulder and elbow without adequate time to recover. Intrinsic non-modifiable risk factors include age, height, sex and previous injury.^6 7^ Impairments of joint range of motion (ROM) except when attributable to humeral torsion,^11–17^ strength^18 19^ and neuromuscular control^20^ are intrinsic modifiable risk factors because their effect may be modifiable through targeted injury prevention programmes.^21^

Changes or side-to-side differences of shoulder ROM result from the repetitive demands of overhead sport,^22–24^ but they may also be a risk factor for injury. In Keller’s systematic review, injured overhead athletes (baseball, handball and tennis) had deficits of shoulder internal rotation, external rotation and total rotation ROM.^16^ Limitations included studies with cross-sectional and retrospective designs, so it is impossible to determine whether the deficits in ROM were present before the injury or were an adaptation to the injury.^16^ Using prospective cohort studies, Bullock *et al*’s^17^ meta-analysis showed that high school baseball players who sustained an in-season shoulder and elbow injuries have less preseason shoulder internal rotation (absolute value: 44°, side-to-side difference: 5°) and total rotation (absolute value 160°, side-to-side difference: 8°) ROM compared with players who did not sustain an injury during the season. However, the authors did not report the magnitude of risk of in-season injuries with an odds or risk ratio for the players with the defined preseason ROM values.^17^ Understanding the strength of the association between risk factors (preseason ROM) and outcomes (injury) is critical to evaluate the ability of preseason ROM to predict risk of injury in overhead athletes, and to design screening and prevention programmes.^21^

The purpose of this systematic review and meta-analysis was to summarise the available evidence, to evaluate the quality of research methods and to characterise the association of preseason shoulder ROM with future risk of shoulder and elbow injuries in prospective cohorts of overhead athletes. We hypothesised that preseason ROM measures of shoulder internal rotation, external rotation, horizontal adduction, shoulder flexion and total rotation have the potential to identify overhead athletes at risk of shoulder and elbow injuries.

## Methods

This review was performed according to the Preferred Reporting Items for Systematic Reviews and Meta-Analyses guidelines.^25^ The review protocol was registered on PROSPERO.

### Data source and search

The following databases were queried for existing evidence (from their inception to September 2018): MEDLINE, Scopus, Embase, Cochrane Library, Cumulative Index to Nursing and Allied Health Literature and SPORTdiscus (via Ebsco). A full-time librarian from the Norris Medical Library of the University of Southern California developed and conducted the search strategy for each database. The search strategies used to query MEDLINE and Cochrane Library are reported in online supplementary appendix A and were adapted for the other databases. Three senior authors with expertise in upper extremity injury in overhead athletes (ES, CAT and LAM) reviewed the list of the included studies to identify studies that were not found through the systematic search of the databases. Further, the reference list of the included studies was hand searched for additional missing studies.

10.1136/bjsports-2019-100698.supp1 Supplementary data

### Study selection

Identified articles were imported in Endnote (Clarivate Analytics, Philadelphia, USA) to screen for duplicates. Afterward, they were exported into Covidence (Covidence systematic review software, Veritas Health Innovation, Melbourne, Australia; available at www.covidence.org) for screening and full-text selection. The following inclusion criteria were used to determine eligibility: (1) inclusion of overhead athletes from Olympic or National Collegiate Athletic Association sanctioned collegiate sports (wide participation), (2) use of preseason measures of ROM; (3) tracked injuries at the shoulder and/or elbow throughout the season, and (4) use of a prospective cohort design. Exclusion criteria included the following: (1) sport does not require overhead repetitive activities; (2) inclusion of contact injuries; (3) lower extremity, spine and hand injuries; and (4) full report not published in English. Studies that assessed humeral retrotorsion were excluded from this review because this physical impairment is not modifiable.^26 27^ Studies that assessed the effectiveness of specific interventions to reduce the risk of shoulder and elbow injuries were excluded from this review.

Two authors (FP and HAP) independently screened the title and abstract to identify relevant studies for the full-text review. A subsample of 100 studies were randomly selected to calculate the agreement between the two reviewers (Cohen’s kappa=0.88, indicating high level of agreement). During both the title and abstract screening and the full-text review, disagreements between the two authors were first discussed. If consensus was not achieved, a third author (LAM) was consulted to make the final decision regarding inclusion or exclusion.

### Assessment of methodological quality

Two authors (PF and HAP) independently scored the methodological quality of each included study using a modified version of the Downs and Black Checklist.^28^ The *Cochrane Handbook* recommends the use of this checklist to appraise non-randomised studies.^29^ The original Down and Black Checklist contains 27 yes/no questions distributed over five sections: reporting, external validity, internal validity (bias and selection bias) and power. Previous systematic reviews that investigated injury risk factors in athletes recommended modifying the Downs and Black Checklist because 6 out of the original 27 questions do not apply to prospective cohort studies.^20 30^ Further, the score of question number 27 (Did the study have sufficient power to detect a clinically important effect where the probability value for a difference being due to chance is less than 5%?) was converted into a dichotomous output (yes=1, the study met the a priori sample target; no=0, the study did not report or did not meet the a priori sample target). The modified checklist used in this study had a maximum score of 21 points, which indicated higher methodological quality. For each article, the raw score and the percentage score [(raw score/21 possible points)×100] was reported. During the assessment of methodological quality, disagreements between the two authors were first discussed. If consensus was not achieved, a third author (LAM) was consulted to make the final decision regarding specific scores.

### Data extraction

One author (FP) extracted the data, which was checked for consistency by a second author (HAP). The following information was obtained: (1) author, (2) year of publication, (3) sport, (4) study population, (4) sample size, (5) sex, (6) age, (7) participants reporting discomfort or injury at baseline evaluation, (8) participants lost to follow-up, (9) number of participants included in the analysis, (10) number of seasons, (11) injury definition, (12) injury tracking, (13) number of injuries and (14) number of injured participants.

### Outcome measures

#### Injury

An injury was defined as any shoulder-related or elbow-related complaint incurred due to competition or training.^31^ Injuries to the shoulder and elbow had to be tracked during the season by healthcare personnel, in-season player interview or self-reported questionnaires.

#### Range of motion

ROM testing procedures, the direction of ROM testing and the side tested were recorded. ROM measurements included two types of variables: (1) absolute ROM of the throwing arm and (2) ROM of the throwing arm expressed as a function of the ROM of the non-throwing arm. The latter often includes a specific ROM cut-off to define the absence or presence of a specific ROM deficit. The type of ROM measure and the cut-off used to identify ROM deficit were extracted for the analysis.

### Data analysis

Only ROM variables that were included as predictors in at least three studies were considered for the meta-analysis. For the studies included in the meta-analysis, odds or risk ratios, confidence intervals and p values were extracted. A random-effect meta-analysis was conducted using the method of Mantel-Haenszel stratified by the direction of ROM and the type of measurement (absolute and deficit). The primary outcome was shoulder and elbow injuries. For all ROM measurements, except for shoulder internal rotation difference, the summarised effect estimate was the odds ratios. For shoulder internal rotation difference, Shanley *et al* 12 reported the risk ratio as effect estimate, while Wilk *et al* 32 33 reported the odds ratio. In order to synthesise the data between these studies^12 32 33^ and to provide an overall estimate, crude odds ratios were converted to crude risk ratios using the formula

$$RR=OR/\left[1-R_{0}+\left(R_{0}XOR\right)\right]$$

where RR is the risk ratio; OR is the odds ratio; and R_0_ is equal to the risk of a positive outcome in the unexposed group. Summary effect estimates and 95% confidence intervals were reported.

I^2^ statistic assessed the heterogeneity of each ROM meta-analysis. Funnel plot and Egger’s test evaluated publication bias and the possibility of a small study effect.

## Results

### Study selection

**Figure 1:**
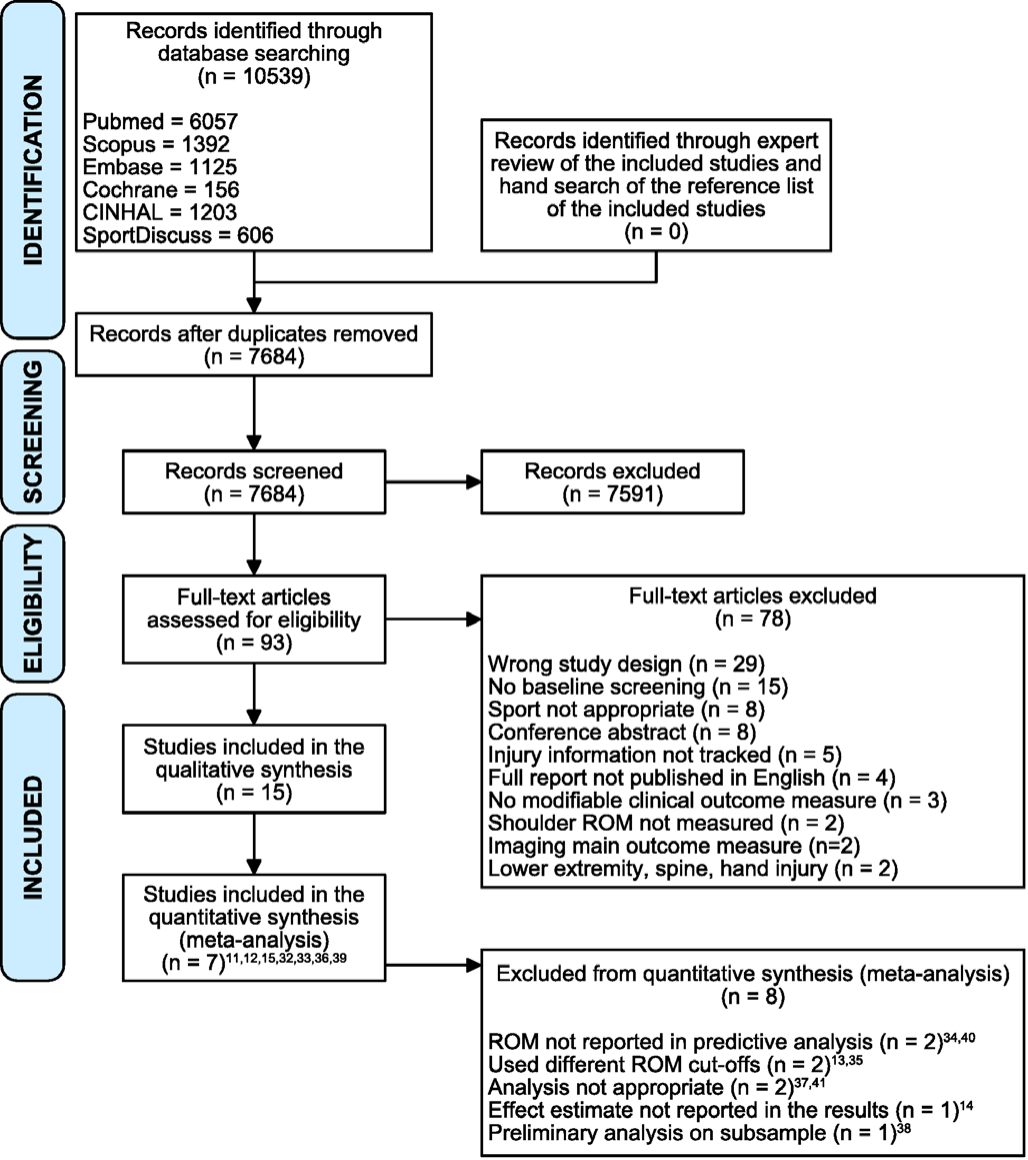
Preferred Reporting Items for Systematic Reviews and Meta-Analyses flow diagram.^25^ Superscript numbers indicate the corresponding reference. ROM, range of motion.

The database search was completed on 22 September 2018. The search identified 10 539 studies (figure 1): 2855 duplicates were removed; 7684 studies were screened; and 93 studies were reviewed in full text. Fifteen studies^11–15 32–41^ met the inclusion and exclusion criteria and were included.

### Study characteristics

Table 1 summarises the characteristics of the included studies. A total of 3314 (female=385) athletes of overhead sports were included in this review, specifically, baseball (n=2471, female=27), softball (n=103, all female), handball (n=535, female=161), tennis (n=65, female=25), volleyball (n=66, female=32) and swimming (n=74, female=37). Six studies included samples of both female and male athletes,^12–14 34 39 40^ but only one considered sex as a covariate in the analysis.^39^ One study^12^ included a cohort of baseball and softball players but reported independent analyses for each sport. One study^35^ included a cohort of youth and adolescent baseball pitchers but reported independent analyses for each age group. A group of authors reported data from the same cohort in two different manuscripts, one that analysed risk factors for shoulder^32^ and one for elbow injuries.^33^ Most of the studies followed up athletes for one competitive season. Nine studies tracked injuries across multiple seasons (range of two to eight seasons).^11 32 33 35 37 38 40 41^ Athletes were re-evaluated at the beginning of each season in seven studies,^11 32 33 35 37 38 41^ while one considered injuries occurring over a 2-year span.^40^ After accounting for athletes evaluated for multiple seasons and lost to follow-up, the total included sample was 3750, specifically baseball (n=3026), softball (n=103), handball (n=428), tennis (n=55), volleyball (n=64) and swimming (n=74).

**Table 1:** Study characteristics

	Sport	Population	Sample	Sex	Age*	Discomfort or injury at baseline	Lost to follow-up	Included in the analysis	Seasons	Injury definition	Injury tracking	Injuries	Injured players
Andersson *et al* 39†	Handball	Upper division	329	168 M 161 F	NR	155 past SH pain 156 SH problem last 7 days 46 substantial SH problem last 7 days	92	267	1	Overuse SH injury (OSTRC score >40)	OSTRC questionnaire (six times during the season)	74	74
Clarsen *et al* 15†	Handball	Professional	206	M	24.0 (4.0)	154 past SH pain 65 SH pain at baseline 23 unable to play due to SH pain	42	161	1	Overuse SH injury‡ (OSTRC score >40)	OSTRC questionnaire (15 times during the season)	108	108
Camp *et al* 11†	Baseball	Professional pitchers	81	M	27.9 (4.5)	NR	4	132 (51 tested multiple seasons)	6	SH or EL injury that caused missing at least one practice or game	Athletic trainer or physician	25 SH 28 EL	50
Oyama *et al* 41	Baseball	High school players	832	M	16.5 (1.2)	NR	NR	832 (tested multiple season NR)	3	SH or EL injury that caused missing/limited exposure in at least one practice or game	Weekly email completed by athletic trainer	25 SH 14 EL 2 Both	41
Sakata *et al* 34	Baseball	Youth players	593	326 M 27 F	9.6 (1.2)	176 previous SH or EL pain (excluded from analysis)	64	353	1	Medial EL pain during throwing with abnormal sonography or pain during clinical assessment	Diaries to record elbow or shoulder pain daily	78	78
Shanley *et al* 35	Baseball	Youth pitchers	47	M	9.9 (1.2)	Exclusion criteria	NR	115 (tested multiple season NR)	3	SH or EL injury that caused missing at least one practice or game	Team athletic trainer or study physical therapist evaluated reported complaint	9 SH 10 EL	33
		Adolescent pitchers	68		14.9 (1.2)							8 SH 8 EL	
Shanley *et al* 12†	Baseball	High school players	143	M	15.8 (1.3)	Exclusion criteria	NR	143	1	SH and EL injury reported by the player to the coach or athletic trainer	Athletic Healthcare System Daily Injury Report form and Simtrack mobility system	16 SH 11 EL	18 baseball
	Softball		103	F	15.6 (1.2)			103					9 softball
Shitara *et al* 36†	Baseball	High school pitchers	132	M	16.3 (0.6)	41 past SH pain 57 past EL pain 17 current SH pain 25 current EL pain	27	105	1	SH or EL injury that caused missing at least 8 days	Injuries reported at medical check-ups	21	21
Tyler *et al* 37	Baseball	High school pitchers	101	M	NR	NR	NR	166 (42 tested multiple seasons)	4	SH or EL injury that caused missing at least one practice or game	Injuries recorded by athletic trainer or physical therapist of each team.	19 SH 9 EL	28
Wilk *et al* 33†	Baseball	Professional pitchers	296	M	24.7 (4.1)	NR	8	505 (126 tested multiple seasons)	8	EL injury requiring placement on the disabled list	Athletic trainers or internet databases	49	38
Wilk *et al* 38	Baseball	Professional pitchers	122	M	25.6 (4.1)	None	NR	170 (38 tested multiple seasons)	3	SH injury causing limited participation or unable to play	Head athletic trainer and team physician	33	30
Wilk *et al* 32†	Baseball	Professional pitchers	296	M	24.7 (4.1)	None	8	505 (126 tested multiple seasons)	8	SH injury requiring placement on the disabled list	Athletic trainers or internet databases	75	51
Hjelm *et al* 40	Tennis	Junior	65	40 M 25 F	15.5 (2.5)	Exclusion criteria	10	55	2	SH or EL injury that caused missing at least one practice or game	Players contacted the investigator if they had an injury	24	24
Forthomme *et al* 14	Volleyball	Professional	66	34 M 32 F	24.0 (5.0)	32 history of SH pain in dominant shoulder	2	64	1	SH pain that caused absence from sport between 1 and 3 weeks	Weekly questionnaire about shoulder pain	3	3
Walker *et al* 13	Swimmers	Competitive	74	37 M 37 F	15.0 (3.0)	Exclusion criteria	1	74	1	SH pain that interfered with competition or training and lasts at least 2 weeks	Weekly diary about injury status	17	17

*Reported as mean (SD).

†Included in the meta-analysis.

‡The authors acknowledged the inclusion of injuries that were acute flare-ups of chronic problems, and long-term problems initially caused by an acute trauma or purely caused by an acute trauma.

EL, elbow; F, female; M, male;NR, not reported; OSTRC, Oslo Sports Trauma Research Centre Overuse Injury Questionnaire; SH, shoulder.

Injury definition varied across studies (table 1). The cumulative shoulder and elbow injury rate in the overall sample of overhead athletes was 17% (666/3750). Divided by sport, the cumulative shoulder and elbow injury rate was 14% (431/3026) for baseball,^11 12 32–38 41^ 9% (9/103) for softball,^12^ 43% (182/428) for handball,^15 39^ 44% (24/55) for tennis,^40^ 4% (3/64) for volleyball^14^ and 23% (17/74) for swimming.^13^

### Risk of bias

The average score on the modified Downs and Black Checklist was 14.9%±2.1% (range 11–18, online supplementary appendix B). Six studies achieved a score of at least 16, which is greater than 75%.^11 13 15 34 36 39^

10.1136/bjsports-2019-100698.supp2 Supplementary data

### ROM measurements

Shoulder ROM directions included flexion, internal and external rotation, and horizontal adduction. Shoulder flexion ROM was measured using a standard goniometer with participants supine, and this methodology was consistent across studies.^11 32 33 40^ Shoulder internal and external rotation ROMs were measured either with a goniometer^11 12 14 32 33 38^ or a digital inclinometer^13 15 35–37 39 41^ with participants supine with shoulder abducted at 90° and elbow flexed at 90°. Horizontal adduction ROM was measured with either a goniometer^11 12 34^ or a digital inclinometer^35 37 41^ with participants supine, according to the procedure described by Laudner *et al*.^42^ Further, nine studies^11 12 15 32–34 36–39 41^ calculated the total rotation of motion by summing internal and external ROMs.

### Preseason screening and in-season shoulder and elbow injuries

Methodological differences prevented including the results of eight studies in the meta-analysis.^13 14 34 37 38 40 41 43^ Three studies^32 33 38^ from the same group of investigators had overlapping data collection time frames: three competitive seasons, from 2005 to 2008,^38^ and eight competitive seasons, from 2005 to 2012.^32 33^ Only the data from the eight competitive seasons were included in the meta-analysis.^32 33^ Softball players were excluded from the internal rotation deficit meta-analysis because none of the nine softball players with at least 20° of shoulder internal rotation deficit sustained an injury.^12^ Table 2 summarises the results excluded from the meta-analysis. Shanley *et al* 35 used a receiver operating characteristics curve to calculate the preseason cut-off of shoulder ROM deficit with the highest sensitivity for risk of shoulder and elbow injuries. In adolescent baseball pitchers, a shoulder internal rotation deficit of at least 13° and a shoulder horizontal adduction deficit of at least 15° were associated with a 5.8 and 4.1 greater risks of shoulder and elbow injuries.^35^ The same analysis did not produce any significant results in youth baseball pitchers.^35^ Shanley *et al* 12 reported that high school baseball players with deficit of shoulder internal rotation ROM greater than 25° are at higher risk (risk ratio=4.8) of injury. In contrast, Tyler *et al* 37 reported that high school pitchers with no internal rotation deficit (side-to-side difference of less than 0°) are at higher risk of shoulder and elbow injuries (risk ratio=4.9) compared with pitchers with a loss of internal rotation of at least 20°. Walker *et al* 13 reported that swimmers with low (<93°) and high (>100°) absolute shoulder external rotations are at risk of a shoulder injury (odds ratios=24.9 and 23.0, respectively) compared with swimmers with shoulder external rotation within 93° and 100°. The odds ratios increased to 32.5 (external rotation <93°) and 35.4 (external rotation >100°) when the statistical model included swimming training distance. Prospective studies in softball,^12^ tennis^40^ and volleyball^14^ players showed that preseason shoulder ROM is not associated with in-season shoulder and elbow injuries.

**Table 2:** Summary of the results from the study that were not included in the meta-analysis

	Shoulder flexion		Shoulder internal rotation		Shoulder external rotation		Shoulder total rotation		Shoulder horizontal adduction	
	Absolute	Deficit*	Absolute	Deficit*	Absolute	Deficit*	Absolute	Deficit*	Absolute	Deficit*
Oyama *et al* 41	…	…	NS†	NS†	NS†	NS†	NS†	NS†	NS†	NS†
Sakata et al^34^	…	…	NI	NI	NI	NI	NS¶	…	NI	NI
Shanley *et al* 35										
Adolescent	…	…	…	>13°: 5.8‡(1.6, 20.9)	…	NS¶	…	NS¶	…	>15°: 4.1‡(1.2, 13.9)
Youth	…	…	…	NS¶	…	NS¶	…	NS¶	…	NS¶
Shanley *et al* 12										
Baseball	…	…	…	≥25°: 4.8** (2.1, 11.3)	…	…	…	NS	…	NS¶
Softball	…	…	…	NS	…	…	…	NS	…	NS¶
Tyler *et al* 37	…	…	…	<0°: 4.9†† (1.0 to 23.3)	…	NS¶	…	NS¶	…	NS¶
Wilk *et al* 38	…	…	…	NS	…	…	…	>5°: 2.5§ (1.1, 5.3)	…	…
Hjelm *et al* 40	NI	NI	NI	NI	NI	NI	…	…	…	…
Forthomme *et al* 14	…	…	NS¶	…	NS¶	…	…	…	…	…
Walker *et al* 13	…	…	…	…	<93°: 24.9‡‡ (2.3, 262.6) >100°: 23.0‡‡ (2.2, 236.8)	…	…	…	…	…

*Range of motion of the throwing arm expressed as a function of the non-throwing arm.

†Analysis compared risk ratio in three groups: below normal, normal and above normal (mean±1 SD used for group definition).

‡Analysis based on the area under the curve of a receiving operating characteristic curve. The odds ratio of the angle cut-off that maximized sensitivity was reported because the authors believed that the cost of participating in a prevention programme is lower than the potential lack of identification of adolescent pitchers at risk of injury.

§Odds ratio

¶Specific effect estimates were not reported in the results.

**Risk ratio.

††Risk ratio for high school pitchers with below-normal internal rotation loss (<0°) compared with pitchers with above-normal internal rotation loss (≥20°).

‡‡Unadjusted odd ratios. Odd ratios adjusted for swim distance (km): <93°: 32.5 (2.7, 389.6) p=0.02 and >100°: 35.4 (2.8, 441.9) p=0.02.

NI, not included in the multivariate predictive analysis; NS, not a significant predictor (odds or risk ratios not reported).

**Figure 2:**
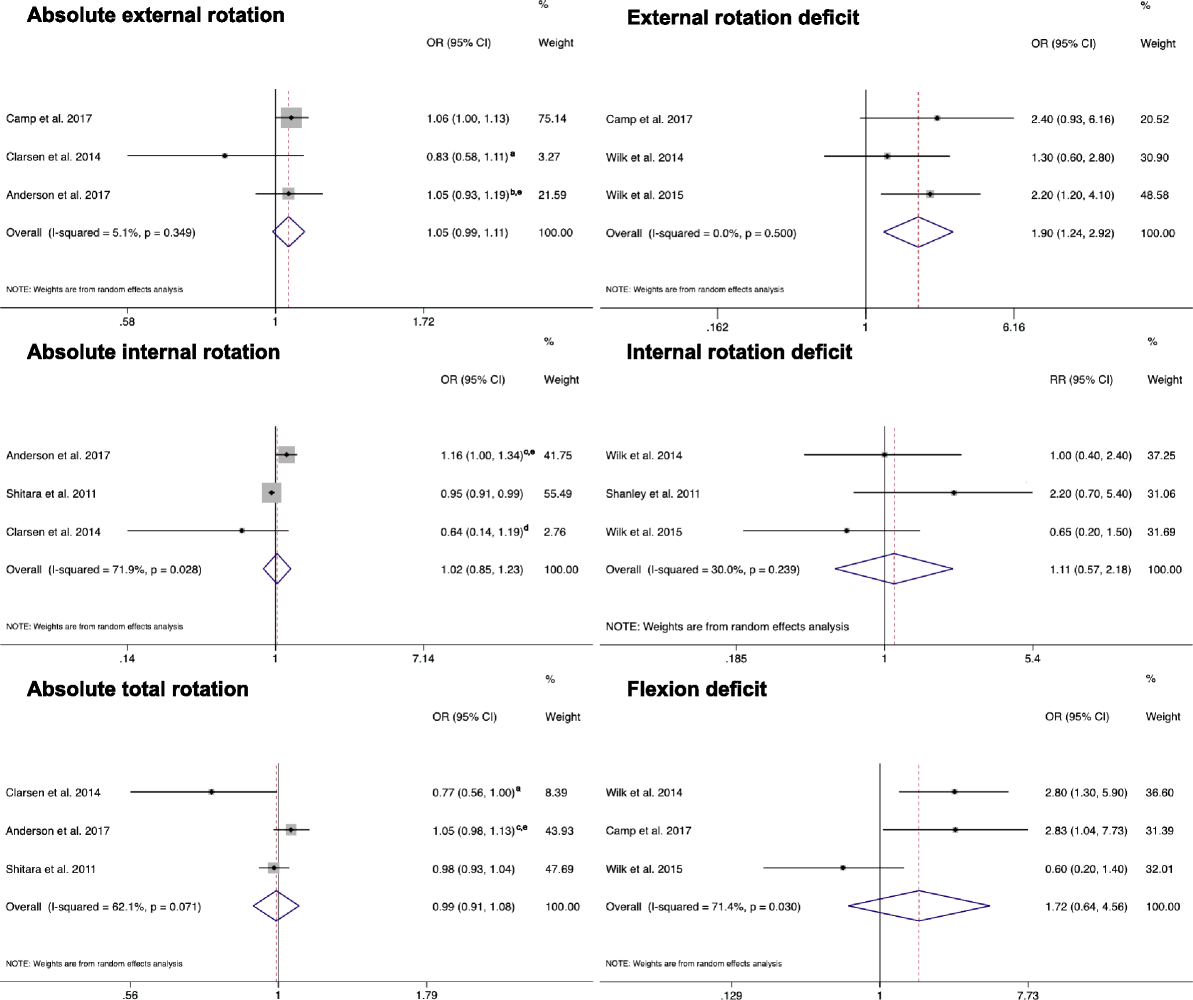
Forest plot indicating the meta-analysis results for all preseason ROM screening and subsequent risk of shoulder and elbow injuries. The summarised effect estimate was the odds ratios for absolute external, internal and total rotation and external and flexion deficit. The summarised effect estimate for internal rotation deficit was the risk ratio. a, adjusted for history of shoulder surgery. b, adjusted for sex and history of shoulder pain during the last season. c, adjusted for sex and shoulder pain at baseline. d, adjusted for player position (back player) and history of shoulder surgery. e, authors reported poor inter-rater and intrarater reliabilities of ROM measurements. CI, confidence interval; OR, odds ratio; ROM, range of motion; RR, risk ratio.

Meta-analyses included data from prospective cohorts of baseball and handball players.^11 12 15 32 33 36 39^ Two studies reported effect estimates that were adjusted based on baseline characteristics (detailed information reported in figure 2).^15 39^ Independent meta-analysis evaluated absolute shoulder ROM of external rotation,^15 36 39^ internal rotation^15 36 39^ and total rotation.^15 36 39^ Other studies measured the absolute value of internal and external rotations,^13 14 40 41^ as well as total rotation,^34 41^ but the methodological differences^13 41^ in the predictive analysis or incomplete results reporting^14 40^ prevented from including these studies in the respective meta-analysis. Only one study measured the absolute value of shoulder flexion and shoulder horizontal adduction.^11^ The results of the meta-analyses indicated that absolute shoulder ROM is not associated with shoulder and elbow injuries (figure 2). A large degree of heterogeneity between studies was found for the absolute value of absolute shoulder internal rotation (I^2^=71.9%, p=0.03) and total rotation (I^2^=62.1%, p=0.07) ROMs.

Independent meta-analyses evaluated shoulder flexion,^11 32 33^ external rotation^11 32 33^ and internal rotation ROM differences.^12 32 33^ Other studies measured external rotation differences^34 36 37 41^ and internal rotation differences,^34–37 41^ but methodological disparities in the predictive analysis,^37 41^ exclusion from multivariate predictive analysis^34^ and different angle cutoffs used to define ROM deficits^35 36^ prevented from including these studies in the respective meta-analyses. Shoulder total rotation and horizontal adduction ROM differences were measured in seven^12 32 33 36–38 41^ and four^11 12 34 35^ studies, respectively. However, the methodological differences in the predictive analysis,^37 41^ the different cut-offs used to define shoulder total rotation or horizontal adduction deficit,^11 12 32 33 35 36 38^ and exclusion from multivariate predictive analysis,^34^ prevented combining the data in meta-analyses. Three side-to-side ROM cut-offs were consistently used across studies to define specific ROM deficits: (1) shoulder flexion: non-throwing arm–throwing arm >5°^11 32 33^; (2) shoulder external rotation: throwing arm–non-throwing arm >5°^11 32 33^ and (3) shoulder internal rotation: non-throwing arm–throwing arm >20°.^12 32 33^ The results of the meta-analyses indicated that the presence of a 5° insufficiency of shoulder external rotation between the throwing and the non-throwing arms (ie, external rotation in the throwing arm was <5° greater than the non-throwing arm) is significantly associated with in-season shoulder and elbow injuries (odds ratio=1.90, 95% confidence interval 1.24 to 2.92, p<0.01; figure 2). The effect estimates of the meta-analysis for external rotation insufficiency did not have substantial heterogeneity (I^2^=0%, p=0.50). In contrast, a large degree of heterogeneity between studies was found for shoulder flexion difference (I^2^=71.4%, p=0.03).

**Figure 3:**
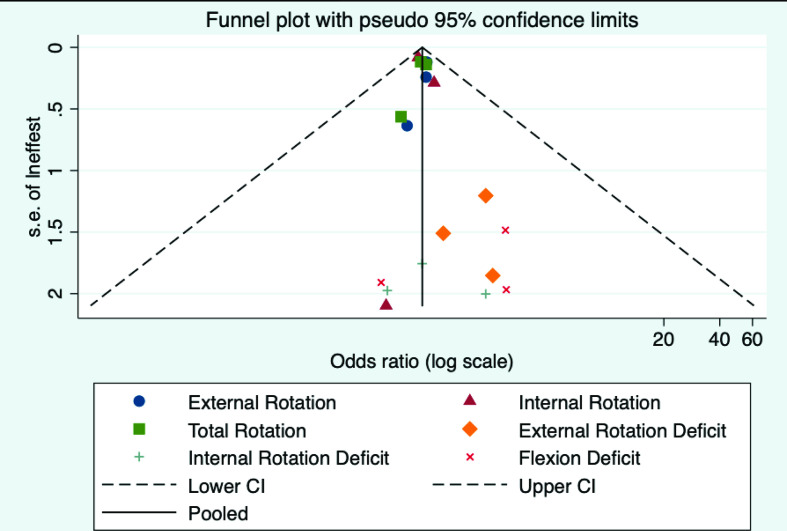
Overall, the plot indicates that all reported studies lie within the 95% confidence limits, suggesting limited reporting bias. The proximity of most studies to the solid black line indicates the null results observed in the bulk of included studies. Some asymmetry is noted, especially for external rotation deficit, indicating likely reporting bias for this exposure.

Overall, the funnel plot was fairly symmetrical and contained within the borders of the funnel, indicating limited publication bias (figure 3). However, there is some evidence of publication bias for external rotation ROM deficit. The funnel plot positive asymmetry suggests that negative or null studies are missing from the published literature. Last, there was no evidence of small study bias (p=0.35).

## Discussion

This systematic review summarised the available evidence, evaluated methodological quality and analysed whether preseason screening of shoulder ROM is associated with the risk of shoulder and elbow injuries in overhead athletes. Overall, we identified 15 prospective cohort studies,^11–15 32–41^ with the majority focusing on baseball. Limited evidence was available for other overhead sports, such as handball, softball, volleyball, swimming and tennis. Female athletes are under-represented, accounting for 12% of the overall sample (34% after removing studies on baseball, which is a male-predominant sports). Only one prospective cohort study of a female-predominant sport of softball^12^ was identified for this review. Our overall hypothesis that preseason shoulder ROM across all overhead athletes identifies those at risk for upper extremity injuries was not confirmed. Summarising the evidence for the meta-analysis was challenging due to the methodological differences between studies. The meta-analysis included three shoulder absolute ROM variables (external, internal and total rotations) and three shoulder ROM deficit (flexion, internal rotation and external rotation). The results of the meta-analysis indicated that professional baseball pitchers were at higher risk of shoulder and elbow injuries when the throwing arm external rotation was not at least 5° greater than the non-throwing arm. Therefore, screening shoulder external rotation ROM may be valuable in professional baseball pitchers.

### Risk of bias

Four studies failed to outline the inclusion and exclusion criteria used to select their sample.^32 33 37 41^ The number of the athletes who were lost to follow-up was clearly described in three studies,^13 34 39^ and five studies reported both the number of athletes that were approached and the number of athletes who agreed to participate.^15 34 36 39 40^ Therefore, some of the included studies may suffer from selection bias. The investigator responsible for preseason measurements was blinded for hand dominance in three studies,^12 35 36^ exposing the remaining studies to potential investigator bias. Only five studies adjusted the analysis for potential confounders.^11 13 15 32 39^ Three studies calculated the required sample size a priori^12 34 36^: two studies met their target sample size^34 36^; one study recruited 82% of the estimated sample due to limited time to perform preseason screening.^12^ Therefore, the majority of the studies may lack sufficient sample size. Three studies did not report the investigator reliability in collecting shoulder ROM.^11 34 37^ Andersson *et al* 39 reported poor inter-rater and intrarater reliabilities for their ROM measurements, which are a critical threat to internal validity that can bias their results.

### Baseball

The risk of shoulder or elbow injuries increased almost twofold if the throwing shoulder of professional baseball pitchers did not have at least 5° greater external rotation compared with the non-throwing shoulder. It is well accepted that the throwing arm of overhead athletes displays greater external rotation ROM compared with the non-throwing arm.^44 45^ Greater shoulder external rotation increases the amount of motion available to develop ball velocity.^46–48^ Professional baseball pitchers with less throwing arm external rotation may employ other strategies, such as dropping their arm slot or allowing their arm to lag behind, to maintain throwing performance, which may place them at higher risk of injury.^49 50^ The fact that less throwing arm shoulder external rotation was associated with shoulder or elbow injury in two independent cohorts of professional baseball pitchers further corroborates the value of screening external rotation ROM in this population.^11 21 32^ The ultimate goal of athlete screening is to reduce their risk of injury by intervening on modifiable risk factors.^21^ Therefore, randomised clinical trials that compare the efficacy of the screening and intervention programme compared with usual training and prevention programmes only are necessary to fully understand the value of screening for shoulder external rotation deficit in professional baseball pitchers.

In contrast, younger baseball pitchers and position players (age 7–18 years) do not consistently display differences in shoulder external rotation in the throwing arm compared with the non-throwing arm.^51^ Although not included in the meta-analysis, four studies^34 36 37 41^ failed to find a positive association between shoulder external rotation difference and subsequent risk of shoulder or elbow injuries in cohorts of junior and high-school baseball players. Bullock *et al* 17 found no absolute differences of preseason shoulder external rotation ROM between a group of high school baseball players who suffered an in-season shoulder or elbow injury and a group who did not. Adaptation of shoulder external rotation ROM may occur over several years of playing and with increased level of performance, which may explain the findings in younger cohorts.

The current meta-analysis indicates that a shoulder internal rotation difference of at least 20° between the throwing and the non-throwing arms is not associated with future shoulder and elbow injuries. The heterogeneity of the studies included in the meta-analysis, which combined professional baseball pitchers^32 33^ and high school baseball players (position players included),^12^ must be considered when interpreting these results. Shoulder internal rotation may not be an important risk factor for professional baseball pitchers.^11 32 33^ In contrast, a recent meta-analysis showed that a preseason side-to-side difference of at least 5° of shoulder internal rotation characterised high school baseball players that sustained an in-season injury.^17^ Screening for 5° side-to-side difference in shoulder internal rotation may generate a high number of false positives, considering that previous studies found that only greater internal rotation difference (favouring the non-throwing arm) carried a higher risk of shoulder and elbow injuries (at least of 13°, adolescent pitchers, and at least 25°, high school baseball players; table 2).^12 35^ Additionally, one study found that high school baseball pitchers with no shoulder internal rotation deficit in their throwing arm have a higher incidence and a higher risk of shoulder and elbow injuries compared with those with at least 20° of shoulder internal rotation differences between the throwing and non-throwing arms.^37^ Thus, unwarranted stretching, which arbitrarily increases the internal rotation on the throwing arm, may also be deleterious for high school baseball players.

A shoulder flexion deficit of at least 5° in the throwing arm is not associated with shoulder and elbow injuries in a homogenous sample of professional baseball pitchers. It is important to note that the anatomical location of the injury was different between the studies included in this meta-analysis. Two studies considered only elbow injuries,^11 33^ while one considered only shoulder injuries.^32^ Based on the reported OR (figure 2), it is unclear why shoulder flexion ROM deficit in the throwing arm would be associated with risk of injury at the elbow, but not at the shoulder. Reduced shoulder flexion may be related to altered latissimus dorsi muscle flexibility. A shoulder flexion deficit of 5° in the throwing arm may result in a lower arm slot during throwing, which has been shown to increase elbow joint stress.^52 53^ Future studies should investigate this potential association.

Bullock *et al* 17 showed that, when measured at preseason, high school baseball players that sustained in-season shoulder and elbow injuries had at least 8° lower horizontal adduction ROM compared with players who did not get injured.^17^ When included in risk analysis, Shanley *et al* 35 found that high school baseball pitchers with a difference of horizontal adduction of at least 15° between the throwing and non-throwing arms were at four times greater risks of shoulder and elbow injuries. Similar findings were not reported in one cohort of professional baseball pitchers,^11^ or in studies including cohorts that combined high school baseball pitchers and position players.^12 34^ Taken together, these findings may indicate that players’ age and position should be considered when screening horizontal adduction ROM.

### Other overhead sports

The evidence available for other overhead sports was limited to two prospective cohorts from the same group of researchers for handball, and one prospective cohort each for softball, volleyball, tennis and swimming.

The two studies^15 39^ that screened absolute shoulder ROM of the throwing arm in handball players found opposite results. Clarsen *et al* 15 reported a small positive association between shoulder total rotation ROM and injury and no association for internal rotation ROM. In contrast, Andersson *et al* 39 reported a small positive association between shoulder internal rotation ROM and injury and no association for total rotation ROM. Caution is warranted when interpreting the results from Andersson *et al* 39 due to the poor inter-rater and intrarater reliability of the ROM measurements. These studies also have some methodological differences that may, in part, explain these contradictory results. Clarsen *et al* 15 included only male handball players, while Andersson *et al* 39 included both male and female. Each study used different confounders to adjust their analysis. Although both studies used the same definition of overuse injury consistent with a non-contact injury mechanism, Clarsen *et al* 15 acknowledged the inclusion of injuries that were acute flare-ups of chronic problems, long-term problems initially caused an acute trauma or purely caused by an acute trauma. The inclusion of acute injuries may also explain the higher injury rate (52%) reported by Clarsen *et al* 15 compared with the study of Andersson *et al* 39 (22%).

While swimmers have different biomechanical demands compared with throwing sports, shoulder pain and injuries are common due to the high repetitions of overhead motion and training volume.^2^ Based on the results of one study,^13^ swimmers with external rotation ROM in the low and high tertiles are at higher risk of shoulder and elbow injuries compared with swimmers whose shoulder external rotation ROM is within 93° and 100° (middle tertile). These results are independent of swimming training distance (table 2).^13^ This ideal external rotation ROM may be protective against shoulder injury, but confirmation of this finding in a second independent cohort of swimmers is needed before making strong recommendations for the use of shoulder ROM screening in this population.^21^

Absolute shoulder ROM or shoulder ROM deficits were not associated with shoulder or elbow injury in high school softball players,^12^ shoulder pain in professional volleyball players^14^ or upper extremity injury in tennis players.^40^

### Limitations

We acknowledge several limitations. Few prospective studies were identified for sports such as handball, softball, tennis, volleyball and swimming. The small number of studies included in each ROM meta-analysis (3 out of 15, 20%) is a significant limitation. With few studies, coverage of the overall effect size is of concern, and one cannot be certain that one large study is not determining the overall effect. Statistical power is limited when the number of studies is low. Lastly, the small number of studies prevented subgrouping within in each meta-analysis.

There was a high degree of heterogeneity among studies for age (youth to adults), position in baseball (pitchers only to combined cohort of pitchers and field players), competition level (competitive to professional athletes) and injury definition (overuse questionnaires, league managed disable lists, combination of symptoms and sonographic findings, symptom duration, and missing time from sport performance, from one game/practice, up to 3 weeks). Combining studies with substantial heterogeneity can mask true differences between studies. It can also lead to combining valid studies with biassed research, producing a biassed overall estimate.

In-season injuries often occur several weeks or months after screening (preseason), and it is possible that the association between screening findings and injuries weakens over time. This is an inherent limitation of all the studies included in this systematic review, as none of the studies reported the time elapsed between screening and injury. Future studies should investigate whether more frequent in-season screenings of factors theorised to relate to injury risk provide better identification of overhead athletes at risk of injury. Most of the included studies did not account for previous injury or exposure (ie, frequency of sport-related activities) in the analysis. This is an important limitation as these factors have been linked to injury and can be potential confounders. The aetiology of injury is multifactorial, and shoulder ROM represents only one risk factor for shoulder and elbow injuries. Thus, the results of this systematic review and meta-analysis should be interpreted with caution.

## Conclusion

Absolute shoulder ROM or shoulder ROM differences do not appear to be consistent risk factors for shoulder and elbow injuries across different overhead athletes. Age, competition level and position should be considered when screening the shoulder ROM of baseball player. Professional baseball pitchers whose external rotation ROM in the throwing arm was not at least 5° greater than their non-throwing arm were twice as likely to sustain in-season shoulder or elbow injuries. Similar findings were not observed in adolescent or high school baseball pitchers. Limited evidence suggested that swimmers with abnormally low or high external rotation are at higher risk of shoulder injuries. Limited evidence suggested that ROM screening may not be effective to identify handball, softball, volleyball and tennis players at risk of shoulder and elbow injuries.

**What is already known**

- The repetitive demands of overhead sport lead to side-to-side changes in shoulder range of motion (ROM), such as increased external rotation and decreased external rotation. However, injured overhead athletes have impairment of shoulder ROM compared with non-injured overhead athletes.

**What are the new findings**

- Professional baseball pitchers whose external rotation ROM in the throwing arm is not at least 5° greater than the non-throwing arm were twice as likely to sustain in-season shoulder or elbow injuries
- Limited evidence: swimmers with external rotation of less than 93° or greater than 100° may be at higher risk of shoulder injuries than swimmers whose ROM is between those limits.
- Limited evidence: ROM screening may not be effective to identify handball, softball, volleyball and tennis players at risk of shoulder and elbow injuries.
