# Supplementary material for: Preseason shoulder range of motion screening and in-season risk of shoulder and elbow injuries in overhead athletes: systematic review and meta-analysis
Source: Br J Sports Med. 2020 Jan 14;54(17):1019–27. doi: 10.1136/bjsports-2019-100698 (PMC7456673; doi:10.1136/bjsports-2019-100698)
Supplement: Supplementary data [file bjsports-2019-100698supp001.pdf]

Appendix A. Search terms used to query PubMed and Cochrane Library databases.

PubMed	Cochrane
1. "Mass Screening" [Mesh]	1. MeSH descriptor: [Mass Screening] explode all trees
2. prevention and control [Subheading]	2. Any MeSH descriptor with qualifier(s): [Prevention & control - PC]
3. "Risk Factors" [Mesh]	3. screening:ti,ab,kw (Word variations have been searched)
4. Screening	4. MeSH descriptor: [Risk Factors] explode all trees
5. Risk	5. risk:ti,ab,kw (Word variations have been searched)
6. Incidence	6. incidence:ti,ab,kw (Word variations have been searched)
7. Risk Factors	7. risk factors:ti,ab,kw (Word variations have been searched)
**8. 1-7/OR**	**8. 1-7/OR**
9. "Athletic Injuries" [Mesh]	9. MeSH descriptor: [Athletic Injuries] explode all trees
10. sport injury	10. sport injury:ti,ab,kw (Word variations have been searched)
11. sports injuries	11. sports injuries:ti,ab,kw (Word variations have been searched)
12. sports injury	12. sports injury:ti,ab,kw (Word variations have been searched)
13. athletic injury	13. athletic injury:ti,ab,kw (Word variations have been searched)
14. athletics injuries	14. athletics injuries:ti,ab,kw (Word variations have been searched)
**15. 9-14/OR**	**15. 8-14/OR**
16. "Upper Extremity" [Mesh]	16. MeSH descriptor: [Upper Extremity] explode all trees
17. Arm	17. Arm:ti,ab,kw (Word variations have been searched)
18. Upper extremity	18. Upper extremity:ti,ab,kw (Word variations have been searched)
19. elbow	19. elbow:ti,ab,kw (Word variations have been searched)
20. forearm	20. forearm:ti,ab,kw (Word variations have been searched)
21. shoulder	21. shoulder:ti,ab,kw (Word variations have been searched)
**22. 16-21/OR**	**22. 16-21/OR**
**23. 8 AND 15 AND 22**	**23. 8 AND 15 AND 22**
