# Supplementary material for: Preseason shoulder range of motion screening and in-season risk of shoulder and elbow injuries in overhead athletes: systematic review and meta-analysis
Source: Br J Sports Med. 2020 Jan 14;54(17):1019–27. doi: 10.1136/bjsports-2019-100698 (PMC7456673; doi:10.1136/bjsports-2019-100698)
Supplement: Supplementary data [file bjsports-2019-100698supp002.pdf]

Appendix B. Critical appraisal of the included studies using the modified version of the Downs and Black checklist.

Study Author	Questions																																Raw score[b]	Percent score[c]
	Reporting											External validity				Internal validity - bias								Internal validity - selection bias							Power			
	1	2	3	4[a]	5	6	7	8[a]	9	10	TOTAL[b]	11	12	13	TOTAL[b]	14[a]	15	16	17	18	19[a]	20	TOTAL[b]	21	22	23[a]	24[a]	25	26	TOTAL[b]	27		
Anderson S, 2017	1	1	1		1	1	1		0	1	7	1	1	1	3		0	0	1	1		0	2	1	1			1	1	4	0	16	76
Camp C, 2017	1	1	1		1	1	1		1	1	8	0	0	1	1		0	1	1	1		0	3	1	1			1	1	4	0	16	76
Clarsen B, 2014	1	1	1		0	1	1		0	1	6	1	1	1	3		0	1	1	1		1	4	1	1			1	1	4	0	17	81
Forthomme B, 2013	1	1	1		0	1	1		0	1	6	1	0	1	2		0	1	1	1		1	4	1	1			0	0	2	0	14	67
Hjelm N, 2012	1	1	1		0	1	1		0	1	6	0	1	1	2		0	1	1	1		1	4	1	1			0	1	3	0	15	71
Oyama S, 2017	1	1	0		1	1	1		0	1	6	1	0	1	2		0	1	1	1		1	4	1	1			0	0	2	0	14	67
Sakata J, 2017	1	1	1		1	1	1		1	1	8	1	1	1	3		0	1	1	1		0	3	1	1			0	1	3	1	18	86
Shanley E, 2015	1	1	1		0	1	1		0	1	6	1	0	1	2		1	1	1	1		1	5	1	1			0	0	2	0	15	71
Shanley E, 2011	1	1	1		0	1	1		0	1	6	1	0	1	2		1	1	1	1		1	5	1	1			0	0	2	0	15	71
Shitara E, 2011	1	1	1		0	1	1		0	1	6	1	1	1	3		1	1	1	1		1	5	1	1			0	1	3	1	18	86
Tyler TF, 2014	1	1	0		0	1	1		0	1	5	1	0	1	2		0	0	1	1		0	2	1	1			0	0	2	0	11	52
Walker H, 2012	1	1	1		0	1	1		1	1	7	1	0	1	2		0	1	1	1		1	4	1	1			1	1	4	0	17	81
Wilk KE, 2014	1	1	0		0	1	1		0	1	5	0	0	1	1		0	1	1	1		1	4	1	1			0	0	2	0	12	57
Wilk KE, 2015	1	1	0		0	1	1		0	1	5	0	0	1	1		0	1	1	1		1	4	1	1			1	0	3	0	13	62
Wilk KE, 2011	1	1	1		0	1	1		0	1	6	0	0	1	1		0	1	1	1		1	4	1	1			0	0	2	0	13	62
**AVERAGE ± St. deviation**											6.2 ± 0.9				2.0 ± 0.8								3.8 ± 0.9							2.8 ± 0.9	0.1 ± 0.4	15.9 ± 2.1	71.1 ± 10.1

[a], Question not applicable to prospective cohort design.

[b], Total possible points: reporting, 8; external validity, 3; internal validity - bias, 5; internal validity - selection bias, 4; raw score, 21.

[c], Calculated as: (raw score/ 21 possible points)*100
